# Dictionaries

**Published:** 1874-01

**Authors:** 


Dictionaries.—Of the indispensable books to a physician's library a good dictionary takes the lead. We know of none which is so well calculated to meet the wants of a physician as is Webster's Unabridged. The definition of medical terms have been made under the eye of an educated physician, and will be found to be correct, and, in most instances, sufficiently extended to meet all the wants which a dictionary is intended to supply.

